# Poor Sleep Quality and Factors Among Reproductive-Age Women in Southwest Ethiopia

**DOI:** 10.3389/fpsyt.2022.913821

**Published:** 2022-07-13

**Authors:** Biruk Bogale, Asrat Wolde, Nuredin Mohammed, Gachana Midaksa, Bayu Begashaw Bekele

**Affiliations:** ^1^School of Public Health, College of Medicine and Health Sciences, Mizan-Tepi University, Mizan Aman, Ethiopia; ^2^Department of Psychiatry, School of Medicine, College of Medicine and Health Sciences, Mizan Tepi University, Mizan Aman, Ethiopia; ^3^Department of Nursing, College of Medicine and Health Sciences, Mizan Tepi University, Mizan Aman, Ethiopia; ^4^Department of Public Health and Epidemiology, Faculty of Medicine, University of Debrecen, Debrecen, Hungary

**Keywords:** poor sleep quality, reproductive age group women, Mizan Aman, Ethiopia, factors

## Abstract

**Background:**

Adequate sleep is vital for physical and mental wellbeing. Sleep-related problems including poor quality of sleep have been increasing throughout the world among reproductive-aged women. Poor sleep quality has been related with number of diseases and health problems However, evidences are scarce regarding poor sleep quality and its associated factors among women of the reproductive age group in Ethiopia.

**Objective:**

To assess sleep quality and associated factors among women of reproductive age group in Mizan Aman town, Southwest Ethiopia.

**Method:**

Community-based cross-sectional study was conducted among 606 reproductive-aged women from 06 November to 20 December 2020, in Mizan Aman town. Data were collected using structured interview administered questionnaires. Pittsburgh Sleep Quality Index (PSQI) was used to assess sleep quality. Multivariable logistic regression was applied using an adjusted odds ratio with a corresponding 95% confidence interval to evaluate the statistical significance of associated factors.

**Result:**

The overall prevalence of poor sleep quality was 71.3%. The late age group of 42–49 (AOR, 95% CI; 1.21 (1.08–5.76), palpable/visible thyroid gland (AOR, 95% CI; 2. 12 (1.08–3.82), current substance use (AOR, 95% CI; 1.76 (1.11–6.10) and having premenstrual syndrome (AOR, 95% CI; 1.86 (1.38–3.12) were significantly associated with poor sleep quality among reproductive age group women.

**Conclusion:**

Significant majority of reproductive age group women faced poor sleep quality. Therefore, screening of sleep patterns among this particular age group is warranted. Moreover, education about sleep hygiene needs to be given considering the identified factors to improve sleep quality.

## Background

Sleep is a natural physiological process that is necessary for an individual's normal physical, emotional, and mental wellbeing (1). Good quality sleep which is characterized by adequate duration, appropriate timing, regularity, and the absence of sleep disturbances or disorders is essential for one's quality of life (2). Sleep quality may reflect different aspects of sleep measures including Total Sleep Time (TST), Sleep Onset Latency (SOL), sleep maintenance, Total Wake Time (TWT), Sleep Efficiency (SE), and sometimes sleep disruptive events such as spontaneous arousal or apnea (3).

Women are increasingly facing sleep-related problems throughout their life cycle. Shreds of evidence showed women are reporting more sleep-related problems when compared to men (3, 4). Hormonal, behavioral, and mood-related issues could worsen the risk among women throughout their life cycle (5, 6). Sleep fragmentation, sleep continuity disturbance, short or long sleep duration, circadian dysrhythmia, and/or hypoxia could all be relevant domains in terms of sleep disturbance among females (7, 8). Reproductive age group women face difficulties related to sleep in premenstrual, menstrual, pregnancy, and during the postpartum period (9). Poor sleep quality is also associated with early pregnancy loss, failed embryo implantation, anovulation and amenorrhea, and even infertility (10, 11).

The magnitude of sleep quality varies across the countries among reproductive-aged group women. A study conducted among young adult women reported that nearly half (45%) of women had poor sleep quality (12). Similarly, 22% of young Korean women experienced poor sleep quality (13). Moreover, the prevalence of poor sleep quality was 28.2% in Nepal (14), 43.7% in the USA (15), and 57% in Canada (16) among reproductive age group women. Multiple factors were identified as predictors of poor sleep quality in this particular age group including socioeconomic and demographic factors, having a history of chronic medical illness, social support, substance use, and history of mental illness (17–22).

Poor quality sleep has been associated with physical, social, economic, and psychological problems (23). Nearly 70% of mental health illnesses such as depression, suicidal ideations, and postpartum psychosis, are caused by sleep pattern abnormalities (24). Moreover, sleep disturbances are also contributed to daytime weakness, tiredness, sluggishness, languid driving, stress, poor impulse control, and pain (25, 26). Poor sleep quality can also be a cause for non-communicable diseases including obesity, hypertension and type 2 diabetes, and could be a primary driver of other health problems (27, 28). Furthermore, Hundreds of billions of dollars a year are spent on direct medical costs associated with health care expenditure and loss of productivity in the work places (29).

Given the deleterious impact of poor sleep quality on health outcomes and wellbeing of reproductive-aged women, this population group should be screened for poor sleep quality regularly (30). Nevertheless, little study pays attention to the quality of sleep among this age group (31). Despite the growing evidences regarding sleep-related problems in a developed country among reproductive-aged women, the pieces of evidence on developing countries including Ethiopia are limited (32). Besides, most of the available evidences are limited to college students, the general population, pregnant and post-partum mothers (32–35). Furthermore, to the best knowledge of the authors, there is no prior study, which examined the sleep quality of reproductive age group women in the study area. Therefore, this study aimed to assess sleep quality and its associated factors among reproductive age group women in Mizan Aman town, southwest Ethiopia.

## Methods and Materials

### Study Design, Setting and Period

A community-based cross-sectional study was conducted among reproductive-age women who live in Mizan Aman town, which is located 586 km southwest of Addis Ababa. Mizan Aman is an administrative capital of Bench Sheko Zone and has five kebeles (lower administrative unit of Ethiopian administration). The town has an estimated population of 52, 210, of which 11,554 are women of reproductive age group (15–49 years). There are one teaching hospital, one health center, five health posts, one university, and one college found in the city. The town has five kebeles and the study was conducted in two selected*kebeles* from 06 November-20 December, 2020.

### Study Population

The source population for this study was all reproductive-aged group women in Mizan Aman town. Reproductive age group women from selected household were the study population and selected reproductive age grouped women from the selected household were the study unit. The study participant was randomly selected from the selected households. Women with serious illness or unable to communicate during the interview were excluded from the study.

### Sample Size Determination and Sampling Technique

The minimum required sample size for this study was calculated using a single population proportion formula. Since there was no previous study regarding sleep quality among reproductive-aged group women in Ethiopia, we assumed a 50% proportion of poor sleep quality, precision level of 5%, 95% confidence interval, and 10% for non-response. Accordingly, the estimated sample size was 422. Hence multistage sampling was employed 1.5 design effect was used and the final sample size was 634.

*Two selectedkebeles* were included in the study. Census was done in the selected kebeles to identify household with reproductive age group women before the data collection. Then, the total sample size (*n* = 634) was allocated proportionally to each selected kebeles based on the number of eligible women. Then systematic random sampling method was employed to select households from those selected *kebeles*. The first house was selected randomly and then every 16^th^ house was enrolled. The sampling interval of the households in each Kebele was determined by dividing the total number of households in the specific kebele by the allocated sample size (10,329/634 = 16). In the case of more than one eligible woman was encountered in the selected household, a lottery method was used to determine which woman would be interviewed.

### Study Variables, Data Collection Tools, and Measurement

The dependent variable of the study was sleep quality (Good or poor). Independent variables were socio-demographic factors such as age, religion, marital status, ethnicity, educational status, income level, employment, and residence, pregnancy, clinical factors such as depression, palpable (visible) thyroid gland, known history of mental illness, and medical illness, and substance use such as alcohol use, khat use, and smoking cigarettes.

To measure poor sleep quality (PSQ) a validated standardized test, the Pittsburgh Sleep Quality Index (PSQI) was used. The PSQI is a 19-item self-report measure designed to measure sleep quality over the past month. It has seven subscales and these subscales are added to determine a global sleep quality score (GSQ). The GSQ score ranges from 0 to 21, the score >5 was labeled as having poor sleep quality and those with PSQI score ≤5 were labeled as having good sleep quality (33). The reliability (Cronbach's alpha) in this study was 0.89.

Depression was assessed by using Patient Health Questionnaire-9 (PHQ-9) and the study participants were considered to have depression if their PHQ-9 score was ≥10 (36).

Current substance use: consuming any substance (Alcohol, Khat and cigarettes smoking) within the last 1 month/30 days (37).

Premenstrual syndrome (PMS): A 10-item tool validated for the assessment of PMS. It has subscales, namely affect, water retention and pain. The total scores were calculated and a score >27 was considered as PMS (38, 39).

Medical illness: Reproductive aged women with chronic medical illness and their diagnosis confirmed in any health institution and assessed by “Yes” or “No” questions.

Palpable (visible): The goiter examination was done and graded according to WHO classification. Grade 0: no goiter (neither visible nor palpable); grade 1: thyroid palpable but not visible; grade 2: thyroid visible with the neck in normal position. Palpable (visible) thyroid gland was considered a grade 1 and 2 thyroid (29).

### Data Quality Control

The data were collected using a standard structured interview administered questionnaire. The English version questionnaires were translated to the local language Amharic. Data were collected by trained three data collectors and two supervisors qualified with a Bachelor of Science degree in psychiatry nursing. The collected data were evaluated for completeness, clarity, and consistency by the supervisor and investigators daily.

### Data Processing and Analysis

The collected data were coded and entered into Epi Data manager version 4.0.2, then, exported to SPSS version 25 statistical software for data analysis. Descriptive statistics like proportion (for categorical variables), mean and SD (for normally distribute continuous variables) were done for different variables. Normality plots (Q-Q plots and/or histograms) were used to check for normality assumptions. Hence, for non-normally distributed variables we used median and interquartile range (IQR) for summary statistics. Bivariable logistic regression was used to identify the associations between the dependent and independent variables. Those variables with a *p*-value of < 0.25 on bivariable logistic regression analysis were transferred to multivariable logistic regression. Interactions and confounders were tested using beta change at cutoff point 20%. Multicollinearity was checked explanatory variables and the variance inflation factor (VIF) was found to be <4. The final model fitness status was checked by Hosmer and Lemeshow's goodness of fit chi-square test (value = 0.79). Adjusted odds ratio (AOR) with a corresponding 95% CI was considered to declare a level of significance for PSQ in multivariable logistic regression analysis.

## Results

### Socio-Demographic Characteristics of the Study Participants

Six hundred six reproductive age group women were enrolled in this study, which makes a 95.6% response rate. The study participants median (IQR) age was 25 (22–36) years and nearly half (45.7%) were in the age group of 24–33 years. Three hundred sixty (59.4%) of the participant were completed secondary or above education and the majority of the participants were located in the monthly income level of ≤1,500 ETB (Table 1).

**Table 1:** Sociodemographic characteristics of a study participant in Mizan Aman town, Southwestern Ethiopia, 2020 (*n* = 606).

**Variables**		**Frequency**	**Percent (%)**
Age	15–23	100	16.5
	24–33	277	45.7
	34–41	182	30
	42–49	47	7.8
Educational status	Illiterate	81	13.4
	Primary school	165	27.2
	Secondary and above	360	59.4
Family Size	≤4	398	65.7
	5–7	132	21.8
	>7	76	12.5
Marital status	Single	174	28.7
	Married	386	63.7
	Divorced	27	4.5
	Widowed	19	3.1
Monthly income status (ETB)	≤1,500 ETB	419	69.1
	>1,500 ETB	187	30.9
Occupational status	Employed	95	15.7
	Farmer	66	10.9
	Student	210	34.6
	Merchant	120	19.8
	Housewife	90	14.9
	Other^a^	25	4.1
Religion	Orthodox	294	48.5
	Protestant	165	27.3
	Muslim	125	20.6
	Others^b^	22	3.6

a *Housemaid and daily laborer*,

b *Catholic and traditional faith, ETB, Ethiopian Birr*.

### Substance Use and Medical Illness

Two hundred forty-one (39.8%) of the participant used substances (Khat, alcohol, or cigarette) at least once in the past 1 month and sixty-four reproductive age group women had a history of mental illness. More than half (51.1%) of the participants had depression (Table 2).

**Table 2:** Substance use and medical conditions among reproductive age group women in Mizan Aman Town, Southwestern Ethiopia, 2020 (*n* = 606).

**Variables**		**Frequency**	**Percent (%)**
Substance use in the past 1month^a^	Yes	241	39.8
	No	365	60.2
Palpable (visible) thyroid gland	Yes	69	11.4
	No	537	88.6
History of mental illness	Yes	64	10.6
	No	542	89.4
Medical illness^b^	Yes	100	16.5
	No	506	83.5
Premenstrual syndrome	Yes	430	71
	No	176	29
Depression	Yes	310	51.1
	No	296	48.9
Pregnancy	Yes	102	16.8
	No	504	83.2

a *Khat, Alcohol & Cigarette*,

b *Hypertension, DM and Asthma*.

### Prevalence of Sleep Quality and Its Component Scores

The overall prevalence of PSQ was 71.3% (95% CI 64–75%). The seven components of sleep quality were assessed using the PSQI and accordingly 153 (25.2%) participants rated their subjective sleep quality very bad and 202 (33.4%) of reproductive women had >7 h of sleep per night. Again, eight participants reported the use of sleep medication during the last month (a month before data collection). Moreover, the habitual sleep efficiency was ≥85% in 20% and <65% in 16.3% of the study participants (Table 3).

**Table 3:** Sleep quality and its component scores among reproductive age group women in Mizan Aman Town, Southwestern Ethiopia, 2020 (*n* = 606).

**Variables**	**Frequency**	**Percent (%)**
Subjective sleep quality		
Very good (0)	197	32.5
Fairly good (1) Fairly bad (2) Very bad (3)	135 121 153	22.3 20.0 25.2
Sleep latency		
≤15 min (0)	168	27.7
16–30 min (1) 31–60 min (2) ≥60 min (3)	133 176 129	22.0 29.0 21.3
Sleep duration		
>7 h (0)	202	33.4
6–7 h (1) 5–6 h (2) <5 h (3)	268 97 39	44.2 16.0 6.4
Sleep efficiency		
≥85%	121	20.0
75–84% 65–74% <65%	189 197 99	31.2 32.5 16.3
Sleep disturbance		
Never (0)	27	4.4
1 time a week (1) 1–2 times a week (2) ≥3 times a week (3)	335 219 25	52.3 36.2 4.1
Use of sleep medication		
Not in the past month	586	96.7
Less than once a week Once (twice) a week 3 or more times a week	11 8 1	1.8 1.4 0.1
Day time dysfunction		
No problem (0)	478	78.9
1–2 times a week (1) 3 times a week (2) > 3 times a week (3)	76 39 13	12.5 6.5 2.1
Sleep quality score		
Good sleep	174	28.7
Poor sleep	432	71.3

### Factors Associated With Poor Sleep Quality

Multivariable logistic regression analysis was conducted with those variables with *p* < 0.25 in bivariable logistic regression analysis. Accordingly, the age of the participant, palpable (visible) thyroid gland, current substance use, and the premenstrual syndrome were independent predictors of PSQ among the study participants (Table 4).

**Table 4:** Bivariable and multivariable logistic regression analysis for factors associated with poor sleep quality among reproductive age group women in Mizan Aman Town, Southwest Ethiopia, 2020 (*N* = 606).

**Variables**	**Poor sleep quality**		**COR at 95%CI**	**AOR at 95% CI**	***P*-value**
	**Yes (%)**	**No (%)**			
**Age**					
15–23	74 (74)	26 (26)	1	1	
24–33	200 (72.2)	77 (27.8)	0.91 (0.67–3.11)	0.82 (0.59–3.67)	0.26
34–41	121 (66.5)	61 (33.5)	0.69 (0.33–5.23)	0.57 (0.47–4.89)	0.33
42–49	37 (78.7)	10 (21.3)	1.3 (1.17–6.45).	**1.21 (1.08**–**5.76)**	0.001^*^
**Palpable (visible) thyroid gland**					
Yes	53 (76.8)	16 (23.2)	2.4 (0.4–1.3)	**2. 12 (1.08**–**3.82)**	0.001^*^
No	379 (70.6)	158 (29.4)	1		
**Family Monthly Income**					
≤1,500 ETB	302 (72.4)	115 (27.6)	1.19 (0.81–2.98)	1.08 (0.72–3.11)	0.38
>1,500 ETB	130 (68.8)	59 (31.2)	1		
**Substance use in the past 1 month**					
Yes	189 (78.4)	52 (21.6)	1.82 (1.22–4.52)	**1.76 (1.11**–**6.10)**	0.002^*^
No	243 (66.6)	122 (33.4)	1	1	
**Having premenstrual syndrome**					
Yes	324 (75.3)	106 (24.7)	1.92 (1.32–2.71)	**1.86 (1.38**–**3.12)**	0.0034^*^
No	108 (61.4)	68 (38.6)	1	1	
**Depression**					
Yes	217 (72.8)	81 (27.2)	1.16 (0.76–4.67)	1.10 (0.71–3.89)	0.16
No	215 (69.8)	93 (30.2)	1		

* *Statistically significant at p < 0.05; COR, Crude Odds Ratio; AOR, Adjusted Odds Ratio*.

Women in the age group of 42–49 [AOR, 95%CI; 1.21 (1.08-5.76)] years were more likely to experience poor sleep quality in comparison to young women in the age group of 15–23 years. The odds of poor sleep quality were 2.12 times higher in women with palpable (visible) thyroid glands than in women with no palpable (visible) thyroid gland. Furthermore, current substance users and women with premenstrual syndrome were 1.86 and 1.76 times more likely to be affected by poor sleep quality, respectively (Table 4).

## Discussion

This study was aimed to determine the prevalence of sleep quality and its associated factors among reproductive age group women. Accordingly, the prevalence of sleep quality is 71.3% (95% CI 64–75). The prevalence observed in this study is comparable with the study conducted in Brazil (66%) (40). However, the prevalence in the current study is higher than studies conducted in Canada (52.8%) (16) and Southeast Texas (43.7%) (15). The possible explanation for this variation could be the socio-economic and socio-cultural differences. This study was conducted during the time the first wave of Covid-19 hit Ethiopia, so there are anxiety and fear of the pandemic, which in turn increase the magnitude of poor sleep quality. Furthermore, being locked down and inability to engage with friends and relatives due to Covid-19 restrictions could also rise the prevalence (41–44). In this study, more than half (51%) of the participants had depression, which is a known risk factor for poor sleep quality and could be another explanation for the observed high prevalence of poor sleep quality (45, 46). Moreover, the study area is around gold mining site where production and problematic substance use are relatively high. Even in our study, 40% of reproductive age group uses substances (Khat, alcohol and cigarette). Substance use especially khat which is an amphetamine type stimulant plant leave produced and consumed locally has been strongly associated with insomnia and could increase the magnitude of poor sleep quality (47, 48).

Women age showed a statistically significant association with poor sleep quality. It indicated that women of premenopausal age are more likely to suffer poor sleep quality. This finding is corroborated by a study conducted in Taiwan were older mothers were three times more likely to have poor sleep quality than younger mothers (49). Premenopausal age has marked with menstrual irregularity due to hormonal alteration and mood changes. The vasomotor symptoms (hot flashes and night sweats) and lower urinary tract symptoms are also common during this period. These changes during premenopausal period further complicates and leads to decline in sleep quality (30, 50).

The current study also showed palpable/visible thyroid gland as a significant factor for poor sleep quality. Women with palpable/visible thyroid glands were at increased odds of having poor sleep quality. Both hyperthyroidism and hypothyroidism has been linked with sleep problems. Hyperthyroidism (overactive) can cause difficulty sleeping due to arousals from nervousness or irritability (51, 52). An overactive thyroid may also lead to night sweats and frequent urges to urinate, both of which can disrupt sleep (52, 53). Similarly, studies showed hypothyroidism also linked with longer onset and shorter duration of sleep caused by difficulty of tolerating cold at night and joint or muscle pain (51, 52). Therefore, these implied the need to screen sleep problems among individuals with palpable (visible) thyroid.

In addition, poor sleep quality is more common among substance users when compared with non-substance users. Substance use including Alcohol, cigarette smoking, and Khat chewing has been identified as a predictor of frequent sleep problems. This finding is consistent with studies done on Norwegian (47) and US adolescents (48) where substance use and sleep problems have a bi-directional effect. Growing evidence shows substance use has a link to mental and physical health problems including sleep disturbance. Sleep-related outcomes of substance use range from extended sleep onset latency (SOL), reduced total sleep time (TST), more nighttime awakenings, and decreased slow-wave sleep (SWS) to rapid eye movement (REM) sleep (54). Educating the effect of substance use on sleep quality needs to be considered in this particular age group.

Moreover, our study showed women with premenstrual syndrome are at increased odds to have poor sleep quality. This is in agreement with studies in South Africa (9), Turkey (55), and Taiwan (56). In support of this finding, menstrual-related hormonal fluctuations may be responsible for this sleep disturbance, which is a common problem during menstruation. Some severe forms of premenstrual syndrome are associated with sleep onset insomnia, frequent night time awakenings, and non-restoration of sleep (57). This calls for the need to give due attention to sleep problems during menstrual period.

The community-based nature of the study and using a validated tool to measure sleep quality among study participants are the strength of this study. However, this study could suffer from social desirability and recall biases.

## Conclusion

This study showed high prevalence of poor sleep quality among reproductive age group women. Age, substance use, premenstrual syndrome, and palpable/visible thyroid gland are identified as factors associated with poor sleep quality. Therefore, due attention needs to be given for this age group women. Moreover, designing effective health education and promotion considering the identified predictors of poor sleep quality could be helpful to decrease the burden of poor quality sleep among reproductive age group women.

## Data Availability Statement

The raw data supporting the conclusions of this article will be made available by the authors, without undue reservation.

## Ethics Statement

Ethical clearance and a supportive letter were obtained from Mizan Tepi University College of Medicine and Health Sciences Ethics Committee (Reference number: 0142/2020) for the commencement of data collection. Written informed consent was obtained from study subjects. For illiterate participant who could not read consent form, the document was orally presented to them or their legally authorized representative as approved by the ethics committee. Again, for minors (age less than 16 years) written informed consent was obtained from parents and/or legal guardian. The participants were informed to decline and stop the interview when they want to do it without any preconditions. Personal identifiers were omitted to maintain the confidentiality of the information. Moreover, the collected data was kept safe throughout the whole data collection and analysis. Those participants diagnosed with depression and goiter were linked to the hospital. This study was conducted following a Helsinki declaration for research ethics.

## Author Contributions

AW designed the study, supervised data, and reviewed the manuscript. BB participated in the design of the study, carried out the statistical analysis, drafted the manuscript, and critically reviewed the manuscript. NM, BBB, and GM participated in statistical analysis and reviewing of the manuscript. All authors read and approved the final manuscript.

## Conflict of Interest

The authors declare that the research was conducted in the absence of any commercial or financial relationships that could be construed as a potential conflict of interest.

## Publisher's Note

All claims expressed in this article are solely those of the authors and do not necessarily represent those of their affiliated organizations, or those of the publisher, the editors and the reviewers. Any product that may be evaluated in this article, or claim that may be made by its manufacturer, is not guaranteed or endorsed by the publisher.
